# Better pulmonary function is associated with greater handgrip strength in a healthy Chinese Han population

**DOI:** 10.1186/s12890-020-1155-5

**Published:** 2020-04-29

**Authors:** Liangmei Chen, Xiaomin Liu, Qian Wang, Linpei Jia, Kangkang Song, Sasa Nie, Yinping Zhang, Dan Cao, Delong Zhao, Zuoxiang Li, Zheyi Dong, Ying Zheng, Shuwei Duan, Xuefeng Sun, Zhe Feng, Guangyan Cai, Weiguang Zhang, Xiangmei Chen

**Affiliations:** 1 Department of Nephrology, The Second Hospital of Jilin University, Changchun, Jilin China; 2 Department of Nephrology, Chinese PLA General Hospital, Chinese PLA Institute of Nephrology, State Key Laboratory of Kidney Diseases, National Clinical Research Center of Kidney Diseases, Beijing Key Laboratory of Kidney Disease, Beijing, China

**Keywords:** Handgrip strength, Pulmonary function, Age, Forced vital capacity, Chinese

## Abstract

**Background:**

Handgrip strength (HGS) has been widely studied in clinical and epidemiological settings, but the relationship between HGS and pulmonary function is still controversial. This study analysed pulmonary function and HGS stratified by sex and age in a healthy Chinese Han population, as well as the associations between HGS and pulmonary function parameters.

**Methods:**

HGS was measured by a Jamar dynamometer and pulmonary function was tested using a portable spirometer. Frequencies and variables are presented as percentages and means ± standard deviations, respectively. Chi-square tests were used for comparisons of categorical variables, and Student’s t-tests or Mann–Whitney U-tests were used for continuous variables. Pearson’s correlation coefficients were used to analyse the normally distributed variables, and Spearman correlation coefficients were used to analyse the non-normally distributed variables. Multivariate linear regression models were employed to explore the relationships between HGS and parameters of pulmonary function. The statistical significance was set at *p* < 0.01.

**Results:**

Cross-sectional data were available for 1519 subjects (59.0% females, 57.9 ± 13.3 years old). Males had higher average HGS than females (40.2 vs. 25.0 kg, *p* < 0.01), as well as better pulmonary function. Both HGS and pulmonary function parameters were significantly inversely correlated with age (r ≤ − 0.30, *p* < 0.01). The maximum value of vital capacity (VC max), forced expiratory volume in 3 s (FEV 3) and forced vital capacity (FVC) were strongly correlated with HGS among the pulmonary function indices (r = 0.72, 0.70 and 0.69, respectively, *p* < 0.001). In the multivariate linear regression analysis, HGS and height were positively correlated, while age and pulse pressure were negatively correlated with HGS. In males, the FVC, VC max and FEV3 increased by 0.02 L, 0.023 L and 0.03 L in per 1 kg increase in HGS, respectively. The HGS coefficients for females were smaller than those for males.

**Conclusions:**

Both pulmonary function and HGS were inversely correlated with age, and better pulmonary function was associated with greater handgrip strength.

## Background

The rapid ageing of the population has become an urgent challenge for China and the world. There were nearly 250 million elderly people (aged> 60 years) by the end of 2018 in China, and it is predicted that the elderly population will grow to approximately 2 billion worldwide by 2050 [1]. Moreover, ageing-associated disorders or diseases are increasing concomitantly.

Pulmonary and musculoskeletal function decline with age, and this phenomenon is not limited to the elderly population. The decline in muscle strength starts at approximately 30 years old and becomes progressive after the age of 65 years [2, 3]. The mean handgrip strength (HGS) declines from 45.5 kg to 23.2 kg for males and from 27.1 kg to 12.8 kg for females between the ages of 25 years and 95 years [4]. Pulmonary function declines even in the absence of pulmonary disease [5, 6], and it is regarded as an independent predictor of mortality.

The correlation between pulmonary function and HGS has been studied [7–12], but this relationship is still controversial. For example, some researchers have claimed that HGS is associated with the forced expiratory volume in 1 s (FEV1) in chronic obstructive pulmonary disease (COPD) subjects [12], while other studies have shown that HGS has no association with lung function but may be associated with the quality of life in COPD patients [8, 11]. More studies have suggested that HGS is positively correlated with parameters of lung function, such as maximum inspiratory pressure [13], forced vital capacity (FVC), FEV1 [14] and peak expiratory flow rate (PEFR) [7]. A cohort study of healthy adolescents revealed that HGS is associated with pulmonary function, while physical activity is not [15].

Most studies on the correlation between pulmonary function and HGS have focused on small samples of individuals with pulmonary disease, stroke, diabetes or other diseases [7–12]. To the best of our knowledge, the association between HGS and pulmonary function has not been investigated in the healthy Chinese Han population.

Therefore, our goal in this study was to analyse pulmonary function and HGS stratified by sex and age in a healthy Chinese Han population, as well as the relationships between HGS and pulmonary function indices.

## Methods

### Subjects and study design

The study was conducted at the Chinese PLA General Hospital in 2016 and recruited volunteers from Beijing, China. All participants received an explanation of the purpose of this investigation and voluntarily provided their consent to participate in this study. All protocols were approved by the Ethics Committee of Chinese PLA General Hospital.

**Fig. 1:**
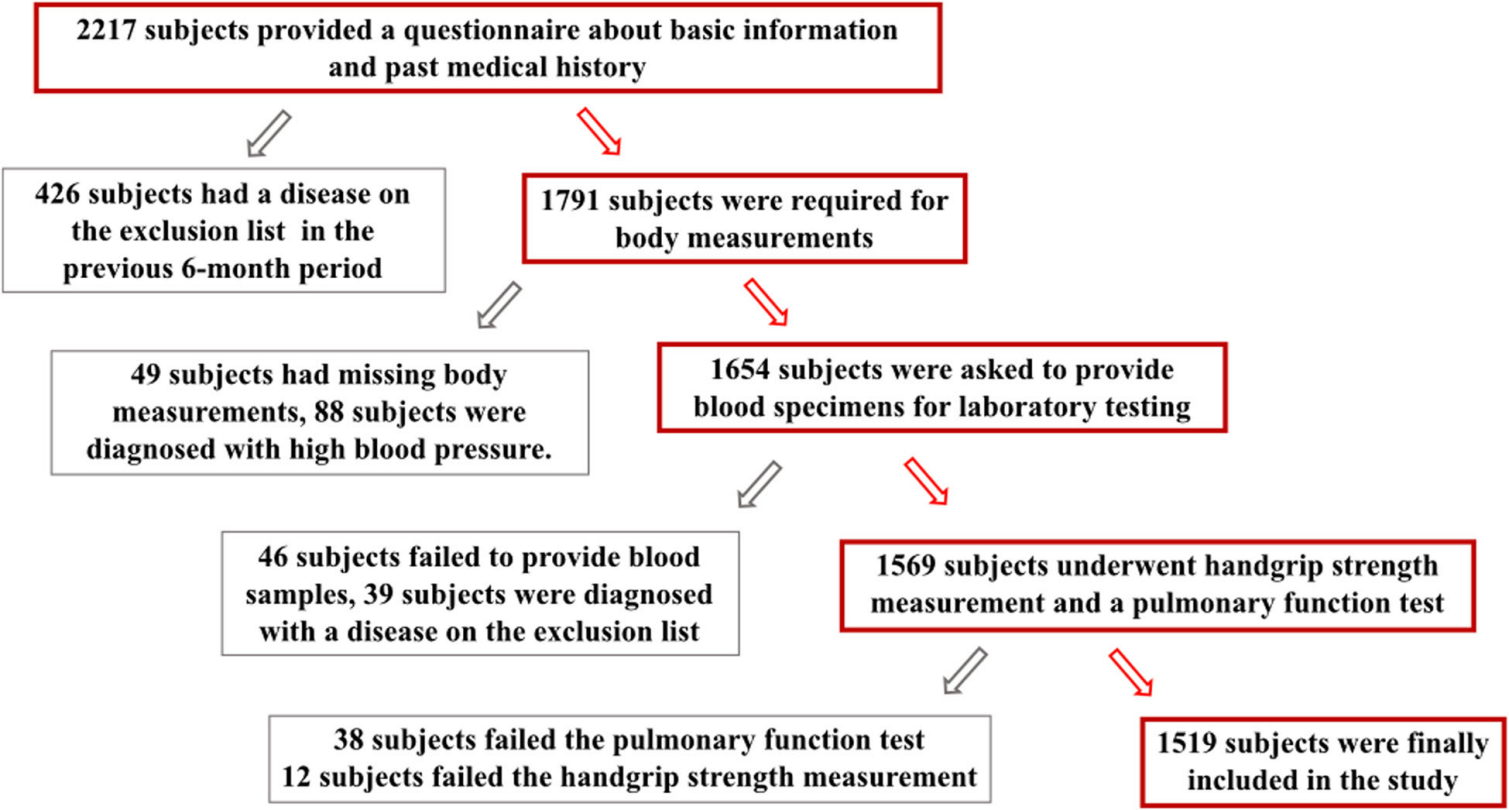
Flowchart for the enrolment of research subjects

In this study, 2217 volunteers were initially recruited (Fig. 1). A total of 598 subjects were excluded by the following exclusion criteria: (a) those with respiratory diseases, such as chronic obstructive pulmonary disease, asthma, bronchiectasis, etc.; (b) those with musculoskeletal disease or rheumatologic disease, such as sarcopenia, fracture, rheumatoid arthritis, etc.; (c) those with obesity (BMI ≥30 kg/m^2^) or metabolic syndrome; (d) those with chronic disease, such as diabetic mellitus, hypertension, chronic kidney disease, etc.; (e) those with one of the following diseases in the previous 6-month period: liver cirrhosis, stroke, myocardial infarction and malignant tumour; and (e) those unable to cooperate with the tests and sample collection.

### Measurements

All participants completed a questionnaire (Additional file 1: Questionnaire) before their medical examination. Information about their medical history, education level, smoking status, drinking and regular exercise was collected from questionnaire provided by the participants. Subjects who exercised at moderate to maximum intensity at least once per week were considered regular exercisers. The questionnaire and examinations were conducted at the Chinese PLA General Hospital. The health examination included body measurements (height, weight, waist circumference, hip circumference, etc.), blood pressure measurements, laboratory tests, HGS measurements and spirometry.

Haemoglobin (Hb), plasma glucose, albumin (ALB), alanine aminotransferase (ALT), triglyceride (TC) and high-density lipoprotein (HDL) cholesterol levels were analysed. Blood pressure was measured using standard mercury sphygmomanometers. HGS (kg) was measured using a Jamar dynamometer (Sammons Preston Rolyan, Bolingbrook, IL) 3 times with the participant’s dominant hand, and the average of the three HGS measurements was used for analysis.

A pulmonary function test was performed by trained medical technicians using a portable spirometer (MasterScreen Pneumo, Hoechberg, Germany) according to the guidelines of the American Thoracic Society/European Respiratory Society [16]. The parameters of pulmonary function are listed in Table 1.

**Table 1:** Index of pulmonary function

Parameters of pulmonary function	Abbreviation
inspiratory vital capacity	VC IN
expiratory vital capacity	VC EX
maximum value of vital capacity	VC max
forced vital capacity	FVC
forced expiratory volume in 0.5 s	FEV 0.5
forced expiratory volume in 1 s	FEV 1
forced expiratory volume in 2 s	FEV 2
forced expiratory volume in 3 s	FEV 3
maximum expiratory flow rate at 25% vital capacity	MEF 25
maximum expiratory flow rate at 50% vital capacity	MEF 50
maximum expiratory flow rate at 75% vital capacity	MEF 75
maximum ventilation volume	MVV

### Statistical analysis

Data were analysed using SPSS 12.0 (SPSS Inc., Chicago, USA). The baseline characteristics are presented as percentages for categorical variables and as the means ± standard deviations for continuous variables. The baseline characteristics of the study participants stratified by gender were compared using chi-square tests for categorical variables and Student’s t-tests or Mann–Whitney U-tests for continuous variables. The statistical significance was set at *p* < 0.01.

Subjects were divided into the younger group (< 60 years) and the older group (≥60 years). HGS and pulmonary function were compared between these two groups. A correlation analysis was employed to analyse the correlation coefficients of HGS and the pulmonary function parameters with age, the relationship between HGS and pulmonary function parameters, as well as other confounding factors affecting pulmonary function. Pearson’s correlation coefficients were used to analyse the normally distributed variables, and Spearman correlation coefficients were used to analyse the non-normally distributed variables. Variables with a correlation coefficient ǀrǀ ≥ 0.30 and *p* < 0.01 were considered to be significantly correlated in our study.

After we identified a linear correlation between HGS and pulmonary function, multivariate linear regression models with VC max, FEV 3 and FVC as dependent variables were conducted to assess the influence of HGS on pulmonary function, with age, height, systolic blood pressure (SBP) and pulse pressure (PP) as independent covariates and “stepwise” as the variable filtering method.

## Results

### Baseline characteristics of the study population

Cross-sectional data were available for 1519 subjects (59.0% females, 57.9 ± 13.3 years old). The demographic and clinical characteristics of the study population are summarized in Table 2. Males had a higher average HGS (40.2 kg) than females (25.0 kg) (*p* < 0.01); compared with females, males also had better pulmonary function (*p* < 0.01).

**Table 2:** General characteristics of the study population

	Males (*n* = 623)	Females (*n* = 896)	Total
Age, years	57.9 ± 13.5	58.0 ± 13.2	57.9 ± 13.3
Handgrip strength, kg	40.2 ± 8.2	25.0 ± 5.7^*^	31.5 ± 10.2
Height, cm	170.9 ± 9.1	159.5 ± 5.1	164.4 ± 9.1
Weight, kg	73.0 ± 12.6	61.4 ± 10.4^*^	66.4 ± 12.9
BMI, kg/m^2^	24.8 ± 4.0	24.1 ± 4.2	24.4 ± 4.2
Waist circumference, cm	91.4 ± 9.1	82.9 ± 11.7	86.6 ± 11.4
Hip circumference, cm	99.8 ± 7.5	97.9 ± 7.3	98.7 ± 7.4
Pulse pressure, mmHg	50.9 ± 10.1	53.0 ± 11.2^*^	52.1 ± 10.8
Hb, g/L	151.9 ± 12.3	133.9 ± 11.4	141.5 ± 14.7
Plasma glucose, mmol/L	5.6 ± 1.7	5.4 ± 1.4	5.5 ± 1.5
Albumin, g/L	45.9 ± 5.0	45.8 ± 4.5	45.8 ± 4.8
Alanine aminotransferase, U/L	24.4 ± 17.6	19.0 ± 15.9	21.3 ± 16.8
Creatinine, μmol/L	83.2 ± 15.0	65.5 ± 11.5^*^	73.1 ± 15.8
TC, mg/mL	4.6 ± 0.9	4.8 ± 1.0	4.7 ± 1.0
HDL, mg/mL	1.3 ± 0.3	1.5 ± 0.4	1.4 ± 0.4
Education, n (%)			
≤ High school	60.4	62.9	61.9
≥ College	39.6	37.1	38.1
Regular exercise, *n* (%)	44.3	47.5	46.2
Drinking, *n* (%)	51.2	25.7^*^	36.1
Cigarette smoking, *n* (%)	32.6	5.7^*^	16.7
Pulmonary function index			
FEV 1 (L)	2.9 ± 0.7	2.2 ± 0.5^*^	2.5 ± 0.7
FEV 2 (L)	3.3 ± 0.7	2.4 ± 0.5^*^	2.8 ± 0.8
FVC (L)	3.3 ± 0.7	2.5 ± 0.5^*^	2.8 ± 0.8
FEV 0.5 (L)	2.3 ± 0.6	1.7 ± 0.4^*^	2.0 ± 0.5
MEF 25 (L/s)	1.3 ± 0.6	1.1 ± 0.5^*^	1.2 ± 0.6
FEV 3 (L)	3.4 ± 0.8	2.5 ± 0.5^*^	2.8 ± 0.8
VC max (L)	3.6 ± 0.7	2.6 ± 0.5^*^	3.1 ± 0.8
VC IN (L)	3.5 ± 0.8	2.6 ± 0.6^*^	3.0 ± 0.8
MEF 50 (L/s)	3.7 ± 1.4	3.0 ± 1.0^*^	3.3 ± 1.2
VC EX (L)	3.4 ± 0.8	2.5 ± 0.6^*^	2.9 ± 0.8
MEF 75 (L/s)	6.0 ± 1.8	4.7 ± 1.2^*^	5.2 ± 1.6
MVV (L/min)	80.3 ± 27.8	65.7 ± 20.3^*^	71.9 ± 24.9

*p < 0.01 for comparison with males. *BMI* body mass index; *Hb* haemoglobin; *TC* total cholesterol; *HDL* high-density lipoprotein; *FEV 0.5/1/2/3* forced expiratory volume in 0.5/1/2/3 s; *FVC* forced vital capacity; *VC IN* inspiratory vital capacity; *VC EX* expiratory vital capacity; *VC max* maximum value of vital capacity; *MEF 25/50/75* maximum expiratory flow rate at 25%/50%/75% vital capacity; *MVV* maximum ventilation volume

### Handgrip strength and pulmonary function were negatively associated with age

The average HGS was significantly higher in the younger group (33.3 kg) than in the older group (27.6 kg) (*p* < 0.01). Pulmonary function was also significantly better in the younger group (*p* < 0.01) (Table 3). In the linear correlation analysis, we found that HGS was significantly correlated with age (r = − 0.30, *p* < 0.01), and the parameters of pulmonary function were strongly negatively correlated with age, especially FEV 1 (r = − 0.55, *p* < 0.01), FEV 2 (r = − 0.53, *p* < 0.01), FVC (r = − 0.50, *p* < 0.01), FEV 0.5 (r = − 0.53, *p* < 0.01), and MEF 25 (r = − 0.60, *p* < 0.01) (Table 3).

**Table 3:** Comparison of handgrip strength and pulmonary function in different age groups and their coefficients of correlation with age

	Younger group (< 60 years, *n* = 725)	Older group (≥60 years, *n* = 794)	r
Handgrip strength	33.3 ± 16.4	27.6 ± 9.4^*^	−0.30
FEV 1 (L)	2.8 ± 0.6	2.2 ± 0.6^*^	−0.55
FEV 2 (L)	3.1 ± 0.8	2.5 ± 0.7^*^	−0.53
FVC (L)	3.1 ± 0.8	2.6 ± 0.7^*^	−0.50
FEV 0.5 (L)	2.2 ± 0.5	1.7 ± 0.4^*^	−0.53
MEF 25 (L/s)	1.3 ± 0.6	1.1 ± 0.5^*^	−0.60
FEV 3 (L)	3.2 ± 0.8	2.6 ± 0.7^*^	−0.47
VC max (L)	3.4 ± 0.8	2.7 ± 0.7^*^	−0.46
VC IN (L)	3.2 ± 0.8	2.7 ± 0.8^*^	−0.44
MEF 50 (L/s)	3.8 ± 1.1	2.8 ± 1.1^*^	−0.50
VC EX (L)	3.1 ± 0.8	2.6 ± 0.7^*^	−0.41
MEF 75 (L/s)	5.7 ± 1.5	4.8 ± 1.5^*^	−0.39
MVV (L/min)	79.6 ± 24.1	64.6 ± 23.4^*^	−0.38

*p < 0.01 for comparison with the younger group;

r is the correlation coefficient between the parameters and age; all correlation coefficients listed are significant at the 0.01 level. Spearman correlation coefficient was used to analyse MVV (with non-normal distribution), and Pearson’s correlation coefficients were used to analyse the normally distributed variables

### Relationship between pulmonary function and handgrip strength

Pearson correlation analysis showed that VC max had the strongest significant correlation (r = 0.72, *p* < 0.001) with HGS for all participants, followed by VC IN, FEV 3 and FVC (r = 0.71; 0.70 and 0.69, respectively) (Table 4). As both VC max and VC IN are parameters of the vital capacity, we selected VC max, FEV 3 and FVC as representatives of lung function to analyse their associations with HGS.

**Table 4:** Correlation coefficients between handgrip strength and parameters of pulmonary function stratified by sex

Parameters of Pulmonary Function	Males (*n* = 623)	Females (*n* = 896)	Total (*n* = 1519)
VC max	0.48^*^	0.51^*^	0.72^*^
VC IN	0.47^*^	0.50^*^	0.71^*^
FEV 3	0.48^*^	0.50^*^	0.70^*^
FVC	0.49^*^	0.50^*^	0.69^*^
FEV 2	0.48^*^	0.52^*^	0.69^*^
FEV 1	0.48^*^	0.46^*^	0.65^*^
VC EX	0.42^*^	0.43^*^	0.65^*^
FEV 0.5	0.45^*^	0.43^*^	0.62^*^
MEF 75	0.34^*^	0.35^*^	0.52^*^
MVV	0.33^*^	0.35^*^	0.41^*^
MEF 50	0.34^*^	0.30^*^	0.40^*^
MEF 25	0.30^*^	0.31^*^	0.30^*^

FEV 0.5/1/2/3: forced expiratory volume in 0.5/1/2/3 s; *FVC* forced vital capacity; *VC IN* inspiratory vital capacity; *VC EX* expiratory vital capacity; *VC max* maximum value of vital capacity; *MEF 25/50/75* maximum expiratory flow rate at 25%/50%/75% vital capacity; *MVV* maximum ventilation volume. ^*^p < 0.001 for the correlation coefficients listed. Spearman correlation coefficients were used to analyse MVV (with non-normal distribution) and MEF 25 (with non-normal distribution only when analysis was stratified by gender). Pearson’s correlation coefficients were used to analyse the normally distributed variables

To identify mixed factors in the relationship between pulmonary function and HGS, we conducted a Pearson correlation analysis between other parameters and pulmonary function (VC max, FEV3 and FVC) (Table 5). HGS, age, height, SBP and PP were significantly correlated with pulmonary function (ǀrǀ ≥ 0.30, *p* < 0.01).

**Table 5:** Correlation coefficients (r) of variables with pulmonary function in males and females

Variables	VC max		FEV3		FVC	
	Males	Females	Males	Females	Males	Females
Handgrip strength	**0.507**	**0.481**	**0.504**	**0.476**	**0.497**	**0.486**
Age	**−0.573**	**−0.604**	**− 0.59**	**− 0.605**	**− 0.611**	**− 0.614**
Height	**0.304**	**0.518**	0.192	**0.508**	**0.281**	**0.504**
Weight	0.226	0.125	0.186	0.161	0.216	0.128
BMI	0.053	−0.065	0.083	−0.053	0.062	−0.055
Waist circumference	−0.017	−0.156	0.02	− 0.185	− 0.013	− 0.155
Hip circumference	0.116	0.027	0.15	0.006	0.115	0.028
SBP	−0.182	**− 0.332**	− 0.118	− 0.278	−0.186	**− 0.334**
DBP	−0.044	− 0.146	0.063	− 0.117	− 0.04	− 0.136
PP	− 0.294	**− 0.38**	− 0.231	**0.325**	**−0.303**	**− 0.385**
Hb	0.240	0.017	**0.303**	0.030	0.263	0.016
Plasma glucose	−0.172	− 0.265	− 0.146	**− 0.356**	− 0.186	− 0.261
Albumin, g/L	0.125	0.087	0.144	0.089	0.148	0.114
Alanine aminotransferase	0.162	−0.101	0.197	−0.102	0.183	−0.079
Creatinine	−0.066	−0.104	0.009	0.019	−0.085	−0.114
TC	−0.014	−0.067	0.059	0.003	0.004	−0.043
HDL	−0.15	−0.005	− 0.202	−0.016	− 0.154	−0.015

The data shown in bold are variables with values of ǀrǀ > 0.30 and *p* < 0.01. Spearman correlation coefficients were used to analyse levels of plasma glucose and alanine aminotransferase (with non-normal distribution in both males and females). Pearson’s correlation coefficients were used to analyse the normally distributed variables.VC max: maximum value of vital capacity; *FVC* forced vital capacity; *FEV3* forced expiratory volume in 3 s; *BMI* body mass index; *SBP* systolic blood pressure; *DBP* diastolic blood pressure; *PP* pulse pressure; *Hb* haemoglobin; *TC* triglycerides; *HDL* high-density lipoprotein.

Then we conducted a series of multivariate linear regression analyses to explore the relationships between pulmonary function (VC max, FVC and FEV3) and four independent variables (HGS, age, height, SBP and PP) (Table 6). The results showed that height and HGS had positive coefficients in multivariate linear regression models, and age and PP had negative coefficients, while SBP was excluded. In males, the FVC, VC max and FEV3 increased by 0.02 L, 0.023 L and 0.03 L in per 1 kg increase in HGS, respectively. The average annual decrease in the FVC, VC max and FEV3 in males was 0.024 L, 0.022 L and 0.028 L, respectively. Compared with males, the effect of age and HGS on pulmonary function was smaller in females, while the effect of height was greater.

**Table 6:** The coefficients of variables in multivariate linear regression models assessing the associations between pulmonary parameters and HGS

Gender	Independent Variables	Unstandardized coefficients				Standardized coefficients (95% CI)			
		Age	HGS	Height	PP	Age	HGS	Height	PP
**Male**	FVC	− 0.024	0.020	0.012	−0.010	−0.449 (− 0.524, − 0.374)	0.225 (0.158, 0.293)	0.148 (0.086, 0.210)	− 0.130 (− 0.182, − 0.065)
	VC max	− 0.022	0.023	0.014	− 0.010	− 0.396 (− 0.468, − 0.324)	0.254 (0.177, 0.320)	0.168 (0.096, 0.228)	−0.134 (− 0.201, − 0.067)
	FEV3	− 0.028	0.030	/	/	−0.464 (− 0.580, − 0.348)	0.303 (0.182, 0.424)	/	/
**Female**	FVC	−0.017	0.018	0.034	−0.005	−0.407 (− 0.479, − 0.359)	0.182 (0.121, 0.233)	0.319 (0.272, 0.375)	−0.097 (− 0.155, − 0.039)
	VC max	−0.016	0.017	0.036	−0.005	−0.393 (− 0.467, − 0.344)	0.175 (0.124, 0.226)	0.337 (0.290, 0.393)	−0.101 (− 0.162, − 0.040)
	FEV3	−0.021	0.019	0.035	/	−0.447 (− 0.532, − 0.362)	0.194 (0.102, 0.286)	0.324 (0.231, 0.407)	/

*HGS* handgrip strength; *PP* pulse pressure; *FVC* forced vital capacity; *VC max* maximum value of vital capacity; *FEV3* forced expiratory volume in 3 s; *VC IN* inspiratory vital capacity. All coefficients listed are significant at *p* < 0.01

## Discussion

Ageing is often accompanied by the functional degradation of multiple organs and systems and the development of ageing-related diseases. The muscular system is characterized by a decrease in muscle mass and a decline in muscle strength during the aging process, and this degradation actually starts at approximately 30 years old [2]. In this cross-sectional study, HGS was negatively associated with age, and the older group had a significantly lower HGS than the younger group. A longitudinal study showed that in people aged 75 years or older, the loss of muscle mass was 0.64–0.70% and 0.80–0.98% per year in females and males, respectively [17]. The loss of muscle strength is 2.5–3% and 3–4% per year in females and males, respectively [18].

Research shows that in Caucasian men after the age of 35 years, lung function begins to decline with increasing age [19], and our study also showed that pulmonary function is negatively correlated with age in a healthy Han Chinese population. Some researchers have reported that ageing might weaken pulmonary function by decreasing the respiratory muscle mass and muscle strength [6, 20].

It is worth mentioning that muscle strength has attracted interest in recent years because a strong and inverse association of muscle strength with all-cause mortality has been confirmed in several populations, such as subjects with cardiovascular disease [21], cancer [22], respiratory disease [23], and chronic obstructive pulmonary disease [24]. HGS, which is a simple, noninvasive and objective marker of muscle strength [25, 26], is widely used in studies of muscle strength.

Moreover, the Prospective Urban Rural Epidemiology (PURE) Study has reported the prognostic value of HGS for all-cause mortality, cardiovascular mortality and cardiovascular disease independent of confounding factors, such as dietary habits, physical activity levels and socioeconomic status [21, 27]. Overall, its simplicity of measurement, portability, low cost and prognostic value make HGS an attractive and important means of evaluating an individual’s overall health in clinical or epidemiological settings [27, 28].

Given the importance of HGS, the Prospective Urban Rural Epidemiology (PURE) Study reported reference ranges of HGS from 125,462 healthy adults in 21 countries, including China. The median HGS in the Chinese population aged 51–60 years reported in the PURE study was 26 kg in women and 40 kg in men [27], which is consistent with our results (women 25.0 kg, men 40.2 kg).

In our study, pulmonary function parameters showed strong, positive correlations with HGS. We further showed that HGS was independently correlated with spirometry. Our results were consistent with previous studies, supporting the conclusion that better respiratory function is associated with greater HGS [13–15, 29–31]. Some studies reported that HGS is a significant predictor of pulmonary function in healthy young adults [32], and some concluded that strength training might improve lung health in adolescents and renal transplant recipients [15, 33].

In addition to HGS, we also found other factors affected lung function, including sex, age, height and PP; these findings were also consistent with those of other studies [7, 9]. Meanwhile, measures of adiposity (such as waist circumference, waist-hip ratio, fat mass, percentage body fat, etc.) and HDL cholesterol were shown to be significantly inversely correlated with pulmonary function in other studies [34, 35].

In this study, we reported the general characteristics of HGS and pulmonary function in a healthy Chinese Han population, as well as the relationship between them: HGS is positively associated with pulmonary function. This study also has several limitations. First, this was a cross-sectional study with a limited sample size and only included individuals belonging to the Chinese Han population. Second, we observed these phenomena in a single centre but failed to explore the intrinsic mechanisms underlying the relationship between HGS and pulmonary function. Further research is needed to identify whether and how HGS can influence pulmonary function.

## Conclusions

In conclusion, HGS and pulmonary function indices were significantly inversely correlated with age, and HGS was positively related to pulmonary function.

## Supplementary information

**Additional file 1.** Questionnaire used in this study.

## Data Availability

The data used to support the findings of this study are available from the corresponding author upon reasonable request.

## Abbreviations

HGS: Handgrip strength
COPD: Chronic obstructive pulmonary disease
PEFR: Peak expiratory flow rate
Hb: Haemoglobin
ALB: Albumin
ALT: Alanine aminotransferase
TC: Triglyceride
HDL: High-density lipoprotein
SBP: Systolic blood pressure
PP: Pulse pressure
VC IN: Inspiratory vital capacity
VC EX: Expiratory vital capacity
VC max: Maximum value of vital capacity
FVC: Forced vital capacity
FEV 0.5: Forced expiratory volume in 0.5 s
FEV 1: Forced expiratory volume in 1 s
FEV 2: Forced expiratory volume in 2 s
FEV 3: Forced expiratory volume in 3 s
MEF 25: Maximum expiratory flow rate at 25% vital capacity
MEF 50: Maximum expiratory flow rate at 50% vital capacity
MEF 75: Maximum expiratory flow rate at 75% vital capacity
MVV: Maximum ventilation volume
