# Supplementary material for: Better pulmonary function is associated with greater handgrip strength in a healthy Chinese Han population
Source: BMC Pulm Med. 2020 Apr 29;20:114. doi: 10.1186/s12890-020-1155-5 (PMC7191764; doi:10.1186/s12890-020-1155-5)
Supplement: Supplementary file 1 — Additional file 1. Questionnaire used in this study. [file 12890_2020_1155_MOESM1_ESM.docx]

**Questionnaire**

**Name:**

**Gender:**

**Age:**

1) What is your education? ({Education}

□ Elementary School □ Junior High School □ High School □ College □Undergraduate □ Master □ Doctor
2) How many years did you go to school ({School time})

______________________

3) What is your marital status? ({Marriage})

□ Unmarried □ Married □ Cohabiting □ Separated □ Divorced □ Widowed
4) Your previous occupation was ({Vocational}):

□ Worker □ Farmer □ Administrative Worker □ Service Industry □ Intellectual

□ Other
5) What are your previous diseases (diagnosed in the hospital)? ({previous disease}) _____________________________________________________________________
6) What are your current health problems? ({Health problems}) _____________________________________________________________________7) What do you think of your health ({health state})?

A. Very bad; B. Bad; C. Not bad; D. Good; E. Very good
8) If you have a disease that can be treated by both Chinese traditional medicine and modern medicine, will you choose traditional Chinese medicine or modern medicine?

A. traditional Chinese medicine; B. modern medicine

9) Do you often go to the hospital to perform physical examination? ({physical examination})

A. Yes; B. No
10) What is your health management approach? ({health management approach})

A. Sports; B. Regulates diet C. Health products D. Drugs E other
11) Do you think life is fun? ({life satisfaction})

A. Absolutely not; B. Less fun; C. Not bad; D. Fun; E. Full of fun
12) What do you think of your quality of life? ({quality of your life})

A. Very bad; B. Bad; C. Not bad; D. Good; E. Very good
13) Can you concentrated? ({concentration})

A. Never; B. Seldom; C. Sometimes; D. Often; E. Always
14) Do you have enough energy in daily life? ({focus})

A. Never; B. Seldom; C. Sometimes; D. Often; E. Always

15) Are you satisfied with the conditions of residence? ({Residence})

A. Very dissatisfied; B. Dissatisfied; C. Not bad; D. Satisfied; E. very satisfied
16) What do you think of your appearance? ({appearance})

A. Very good; B. Good; C. Not bad; D. Bad E. Very bad
17) Do you have enough money? ({Money})

A. Totally not; B. Not enough; C. Mostly enough; D. Enough; Completely enough
18) Do you have time for leisure activities? ({leisure})

A. Never; B. Seldom; C. Sometimes; D. Often; E. Always
19) What is your ability to act? ({activity})

A. Very good; B. Good; C. Not bad; D. Bad E. Very bad
20) Are you satisfied with your sleep? ({sleep satisfaction})

A. Very dissatisfied; B. Dissatisfied; C. Not bad; D. Satisfied; E. Very satisfied
21) Are you satisfied with your daily abilities? ({capacity})

A. Very dissatisfied; B. Dissatisfied; C. Not bad; D. Satisfied; E. Very satisfied

22) Are you satisfied with your work ability? ({Ability to work})

A. Very dissatisfied; B. Dissatisfied; C. Not bad; D. Satisfied; E. Very satisfied
23) Are you satisfied with your relationships? ({relationship})

A. Very dissatisfied; B. Dissatisfied; C. Not bad; D. Satisfied; E. Very satisfied

24) Are you satisfied with your sex life? ({sexual})

A. Very dissatisfied; B. Dissatisfied; C. Not bad; D. Satisfied; E. Very satisfied

25) Are you satisfied with yourself? ({own satisfaction})

A. Very dissatisfied; B. Dissatisfied; C. Not bad; D. Satisfied; E. Very satisfied

26) Can you get satisfactory help from your friends? ({friend to help})

A. Very dissatisfied; B. Dissatisfied; C. Not bad; D. Satisfied; E. Very satisfied

27) Are you satisfied with the convenience of getting health care services? ({Health care services})

A. Very dissatisfied; B. Dissatisfied; C. Not bad; D. Satisfied; E. Very satisfied

28) Are you satisfied with your traffic situation? ({traffic})

A. Very dissatisfied; B. Dissatisfied; C. Not bad; D. Satisfied; E. Very satisfied

29) Do you have negative feelings such as depression, despair, anxiety, and depression? ({Negative feelings})

A. Never; B. Seldom; C. Sometimes; D. Often; E. Always

30) Does family friction affect your life? ({Family friction})

A. Never; B. Seldom; C. Sometimes; D. Often; E. Always
31) How is your appetite? ({Appetite})

A. Very good; B. Good; C. Not bad; D. Bad E. Very bad

32) Based on all aspects of your life quality, give a total score, how many points do you score? (Out of 100 points)

33) How many children do you have ({children})
34) How many people do you live with ({Residence})
35) Whether it is empty nest ({empty nest})

□ Yes □ No
36) Whether to live alone ({Alone})

□ Yes □ No
37) Do you smoke cigarette? ({smoke})

□ Ex-smoker; □ Current smoker; □ Never
38) How many cigarettes to suck per day ({cigarettes})

39) How many years have you sucked ({smoked for many years})

40) Are there any fixed hobbies ({Fixed hobby})

□ Yes □ No
41) Your hobby is ({interests}): ___________________
42) How often do you participate in physical exercise? ({Physical excercise})

A every day; B 2-3 times a week; C once a week; D occasionally; E never

43) How do you exercise?

A. Weak intensity (walking, jogging, etc); B. Moderate intensity (running, swimming, balls, etc.); C. Maximum intensity (anaerobic exercise needing equipment, resistance exercise like lift weights and push-up, etc.)
44) Do you eat regular meals at regular intervals? ({meals})

A. timed and quantitatively; B. timed but not quantitatively; C. quantitatively but untimed; D irregularly and not quantitative
45) Do you eat breakfast? ({breakfast})

A. Daily; B. Sometimes; C. Rarely; D. Never.
46) Where do you usually eat breakfast? ({Breakfast place})

A. At home; B. Company; C. Roadside stall; D. Rarely eat.
47) What do you usually eat for breakfast? ({breakfast})

A. Congee, noodles, buns, milk, eggs; B. Biscuits, bread, milk drinks; C. Fast noodles, fast food D. Fried food.
48) How do you eat lunch? ({lunch})

A. Eat at home; B. Order at the canteen or restaurant; C. Bring meals; D. Fast food.
49) How do you eat dinner? ({Dinner})

A. Eat at home; B. Eat in a restaurant; C. Fast food; D. Only eat vegetables and fruits
50) Do you have the habit of eating supper? ({supper})

A. Never; B. Seldom; C. Sometimes; D. Often; E. Always
51) Do you often eat fast foods? ({Fast Food})

A. often; B occasionally; C never.
52) What do you think of the roadside stalls? ({street vendors})

A. no problem for everyone to eat; B. I like roadside stalls and I often eat; C is unhygienic and I seldom eat; D strongly against roadside stalls
53) Will your mood affect your diet? ({diet and mood})

A. Often; B Sometimes C. Never; D Depending on my mood.
54) Do you wash your hands before meals? ({Wash your hands before meals})

A. Often; B Sometimes C. Never.

55) Are you overeating? ({overeating})

A. Often; B Sometimes C. Never.

56) How fast do you think you are eating? ({eating speed})

A. Fast; B Normal; C Slow.
57) Will you deliberately diet in order to lose weight? ({deliberately dieting})

A. Often; B Sometimes C. Never;

58) Do you chat while eating? ({dinner chat})

A. Often; B Sometimes C. Never;

59) Do you have a habit of picky eating? ({picky eaters})

A. Yes; B. No

60) What snacks do you like to eat? ({Snacks})

A. Nut; B. Biscuit or snack; C. Dried meat or dried fish; D. Preserved fruit, puffed food E. Never eat
61) When do you eat snacks every day? ({eat snacks})

A. Between meals; B. No limited; C. During meals; D. Eat before bed; E Never eat.
62) What is the taste of your diet? ({Food flavors})

A. Light; B. Sweeter; C. Salty; D. Greasy.
63) What is your staple food structure? ({staple structure})

A. Rice and noodles mainly, less coarse grain potatoes; B. Rice white noodles and coarse grain potatoes, basically equal amounts; C. Coarse grains, potatoes mainly, a small amount of rice white noodles; D. Only eat rice white noodles.
64) How much can you eat on a daily basis? ({staple food})

A. 400-500g; B.250-400g; C. 100-250g; D. 100g or less
65) How often have you eaten coarse grains (corn, millet, sorghum, oats, buckwheat, etc.)?

A. Eat every day; B. More than three times a week; C. Less than twice a week;

D. Seldom
66) How do you eat soy products (soybean milk, tofu, bean sprouts, dried beans, etc.) ({Bean})?

A. Eat every day; B. More than three times a week; C. Less than twice a week;

D. Seldom
67) What is your intake of milk and milk products (fresh milk, pure milk, yogurt, milk-containing beverages, milk powder, cheese)? ({milk})

A. Eat every day; B. More than three times a week; C. Less than twice a week;

D. Seldom
68) What is your daily intake of milk and dairy products (fresh milk, pure milk, yogurt, milk-containing beverages, milk powder, cheese)? ({Dairy intake})

A. More than 300g; B. 100- 300g; C. Less than 100g; D. Seldom intake.
69) Do you often eat eggs? ({eggs})

A. Eat every day B. More than 3 times a week C. Less than twice a week D. Seldom

70) Do you pay attention to the combination of meat and vegetables? ({meat and vegetables})

A. Take meat as the main B, take vegetarian as the main C relatively balanced D. Don't pay attention
71) Do you often eat animal food (pork, beef, lamb)? ({animal food})

A. Eat every day B. More than three times a week C. Less than twice a week D. Seldom
72) Are you a vegetarian? ({Vegetarian})

A. No, I eat lean meat and fish every day; B. Yes, but there is a corresponding daily intake of egg or milk; C. No, I like meat, especially fat meat; D. Yes, totally vegetarian.
73) The consumption of animal offal (liver, kidney, stomach, intestine) ({animal offal}) A. Seldom; B. Less than 3 times per month; C. More than 3 times per week; D. Everyday
74) You eat fatty meat or meat oil ({lard})

A. Seldom; B. Less than three times a week; C. More than three times a week; D. Every day
75) You eat fish, shrimp, crab and other aquatic products ({aquatic})

A. Eat every day B. More than three times a week C. Less than three times a week D. Basically do not eat
76) Do you often eat fish from the deep sea (such as tuna, croaker, etc.)? ({deep-sea fish})

A. Eat regularly, 4-5 times a week; B. 2-3 times a week; C. Rarely eat; D. Never eat
77) What is your daily consumption of fresh vegetables? ({Vegetables})

A. 300g or more; B. 200-300g; C. 100-200g; D. 100g or less
78) Do you eat fruit? ({fruit})

A. Daily; B. Sometimes; C. Rarely; D. Never.

79) Do you like to eat yellow, red and purple colored vegetables such as carrots, peppers and tomatoes? ({Vegetables})

A. Frequently; B. 2-3 times a week; C. Rarely; D. Never

80) What is your daily consumption of fresh fruit? ({Fresh fruit})

A. 200g or more; B. 100-200g; C. 100g or less; D. Never
81) When do you eat fruit every day? ({eat fruit})

A. Between meals; B. before meals; C. Eat after meals; D. Eat little or no food
82) Do you have a habit of drinking water every morning? ({morning drink})

A. Yes; B. No

83) Do you drink water or beverages while dining? ({Dining drink})

A. often; B. occasionally; C. never
84) What is your daily water consumption? ({Daily water consumption})

A. More than 1500 ml; B. 1000-1500ml; C. 500-1000ml; D. less than 500 ml
85) What kind of water do you drink? ({Kind of Water})

A. Mineral water; B. Tap water (white boiling water); C. Tea water; D. Pure water.
86) Your drinking habit is ({drinking habits})

A. Get up early and drink a cup of warm water on an empty stomach; B. Drink in the morning and afternoon; C. Irregularly D. Drink only when you feel thirsty
87) What kind of beverage do you often drink? ({Drink})

A. Pure fruit juice; B. Non-carbonated sugary drinks; C. Coffee; D. Carbonated drinks E. Never drink.
88) Do you have a habit of drinking soup or porridge? ({soup or porridge})

A. Drink every meal; B. Once a day; C. More than three times a week D. Less than twice a week
89) When do you usually drink soup or porridge? ({soup or porridge})

A. Drink before meals; B. Drink while eating; C. Rarely drink; D. Drink after meals
90) Do you pay attention to the daily dosage of salt? ({Salt}) What is the dosage?

A. Yes, 6g or less; B. Yes, 6-12g D. Yes, 12g or more; C. Pay no attention

91) What is the daily amount of cooking oil in your family? ({oils amount})

A. 25 g or less; B.25-35 g C. 35 g or more D. not sure, add with taste
92) What oil do you use in your home? ({oil used})

A. Salad oil, blended oil; B. Vegetable oils such as rapeseed oil, soybean oil, etc.; C. Animal oils such as lard or tallow; D. Not fixed

93) The cooking method commonly used in your home cooking is ({cooking})

A. Cold, steamed B. Quick fried C. Boiled, stewed, braised D. Fried
94) Do you have the habit of eating preserved or fried food? ({Pickled})

A. No, never eat; B. Eat occasionally; C. Often eat; D. Eat almost every day

95) Do you drink? (Drinking)

A. Nondrinking; B. Occasional drinking; C. Frequently drinking
96) Do you often get drunk? ({Drunk})

A. Never; B. Seldom; C. Sometimes; D. Frequently; E. Always
97) The transportation you use to commute to work every day ({Vehicle})

A. Walking or by bicycle; B. Public transportation; C. Driving; D. Not fixed

98) Are you often in a state of high stress at work? ({work pressure})

A. No; B. Occasionally; C. Frequently; D. Every day
99) How many hours do you work every day? ({work time})

A. 6 hours or less; B. 6-8 hours; C. 8-12 hours; D.> 12 hours; E. Retired
100) Do you often go out for entertainment? ({socializing})

A. Occasionally; B. Never; C. Often; D. Every day
101) How often do you perform routine medical examinations? ({Physical examination})

A. Twice a year; B. Once a year; C. Occasionally; D. Never.
102) How do you feel about your health condition? ({health condtion})

A. good; B. average; C. poor; D. not sure

103) How often does your stool excrete? ({stool})

A. more than twice a day; B. twice a day; C. Once a day; D. Once 3-5 days;

E. Once more than 5 days
104) Do you have symptoms of calcium deficiency (backache, backache, cramps, joint pain)? ({calcium deficiency})

A. Never; B. Occasionally; C. Often; D. Every day
105) Do you have the habit of supplementing some essential nutrients? ({Supply nutrition})

A. Every day; B. Occasionally; C. Never

106) What nutritional supplements have you taken? ((nutrition)

A. Calcium, zinc and other minerals; B. Vitamins; C. plant extracts; D. Never used
107) Did you complete this questionnaire with the help of others? ({done by yourself})

□ Yes □ No
108) How long did it take you to complete this questionnaire? ({questionnaire})

___ minutes
